# Tumor growth suppression using a combination of taxol-based therapy and GSK3 inhibition in non-small cell lung cancer

**DOI:** 10.1371/journal.pone.0214610

**Published:** 2019-04-10

**Authors:** Linda O’Flaherty, Steven D. Shnyder, Patricia A. Cooper, Stephen J. Cross, James G. Wakefield, Olivier E. Pardo, Michael J. Seckl, Jeremy M. Tavaré

**Affiliations:** 1 School of Biochemistry, Medical Sciences Building, University of Bristol, Bristol, United Kingdom; 2 Institute of Cancer Therapeutics, University of Bradford, Tumbling Hill, Bradford, United Kingdom; 3 Wolfson Bioimaging Facility, Medical Sciences Building, University of Bristol, Bristol, United Kingdom; 4 Biosciences / Living Systems Institute, College of Life and Environmental Sciences, University of Exeter, Exeter, United Kingdom; 5 Department of Oncology, Hammersmith Campus, Cyclotron Building, London, United Kingdom; Virginia Commonwealth University, UNITED STATES

## Abstract

Glycogen synthase kinase-3 (GSK3) is over-expressed and hyperactivated in non-small cell lung carcinoma (NSCLC) and plays a role in ensuring the correct alignment of chromosomes on the metaphase plate during mitosis through regulation of microtubule stability. This makes the enzyme an attractive target for cancer therapy. We examined the effects of a selective cell-permeant GSK3 inhibitor (CHIR99021), used alone or in combination with paclitaxel, using an *in vitro* cell growth assay, a quantitative chromosome alignment assay, and a tumor xenograft model. CHIR99021 inhibits the growth of human H1975 and H1299 NSCLC cell lines in a synergistic manner with paclitaxel. CHIR99021 and paclitaxel promoted a synergistic defect in chromosomal alignment when compared to each compound administered as monotherapy. Furthermore, we corroborated our *in vitro* findings in a mouse tumor xenograft model. Our results demonstrate that a GSK3 inhibitor and paclitaxel act synergistically to inhibit the growth of NSCLC cells *in vitro* and *in vivo* via a mechanism that may involve converging modes of action on microtubule spindle stability and thus chromosomal alignment during metaphase. Our findings provide novel support for the use of the GSK3 inhibitor, CHIR99021, alongside taxol-based chemotherapy in the treatment of human lung cancer.

## Introduction

It is well established that glycogen synthase kinase-3 (GSK3) phosphorylates a wide range of protein substrates which, in turn, regulate a plethora of cellular processes including the control of cell metabolism, differentiation, proliferation and apoptosis [1–5]. Considering this multi-functionality, therefore, it is not surprising that GSK3 has been implicated in several diseases ranging from schizophrenia, neurodegeneration and diabetes, to cancer [6–8].

The role of GSK3 in cancer appears to be cancer type specific [9]: in some tumor types it acts as a tumor suppressor [10, 11] while in others it appears to be a tumor promoter [12–17]. Related to the latter, increased expression and/or activity of GSK3 has been observed in colorectal cancer [12], osteosarcoma [18], renal cell carcinoma [19] and, by ourselves, in non-small cell lung cancer (NSCLC) [20]. Interestingly, it has been reported that tumor cell resistance to chemotherapy and radiotherapy can be overcome by either direct inhibition of GSK3 [21] or targeting of the AKT/GSK3β pathway [22]. Therefore, inhibition of GSK3 may be an appropriate therapeutic intervention in several cancer types where GSK3 has a tumor promoting role [23]. In support of this, there have been numerous studies describing the anti-proliferative effects of small molecule inhibitors of GSK3 in the following tumor cell types: pancreatic [24], ovarian, [14, 25] mixed lineage leukemia [26], glioma [27] and NSCLC [16, 28–30].

In NSCLC, it was initially suggested that GSK3 activity was reduced on the basis of an observed increase in phosphorylation of the inhibitory N-terminal serine phosphorylation site on the enzyme (Ser21 on GSK3α and Ser9 on GSK3β)[11]. However, while we confirmed that GSK3 Ser21/9 phosphorylation was indeed increased in NSCLC tumor tissue compared to that in the surrounding patient-matched normal lung tissue, we found that this inhibitory effect was counteracted by the over-expression of the enzyme. We previously demonstrated that this led to an overall net increase in protein kinase activity rather than the decrease that was originally assumed [20] This is of important clinical relevance as it has been suggested that increased expression of GSK3β in NSCLC is associated with poor patient prognosis [16]. In support of GSK3 inhibition as a viable therapeutic strategy, a recent first-in-human phase I trial demonstrated that intravenous administration of the GSK3 inhibitor, LY2090314, in combination with pemetrexed and carboplatin was tolerated at a safe dose with mesothelioma and NSCLC patients showing the most promising reduction in tumor size from baseline [31].

We have previously reported that inhibition of GSK3 by CHIR99021, a highly selective GSK3 inhibitor [32], stabilises spindle microtubules in HeLa cells, resulting in misalignment of chromosomes on the metaphase plate and defective chromatin segregation during mitosis [33]. Paclitaxel, a chemotherapeutic agent extensively used in doublet therapies against NSCLC, promotes apoptosis via stabilisation of microtubule structures and disruption of normal chromatin segregation [34, 35]. Therefore, we set out to compare the effects of paclitaxel and CHIR99021, on NSCLC cell growth in culture and in a mouse tumor xenograft model.

Here, we report that by combining paclitaxel treatment with CHIR99021 we observe a striking synergistic effect of the compounds on reducing NSCLC tumor cell growth both in an *in vitro* model and in an *in vivo* tumor xenograft. Our findings provide promising support for the use of the GSK3 inhibitor, CHIR99021, alongside taxol-based chemotherapy in the treatment of human lung cancer.

## Methods and materials

### Ethics statement

This investigation was conducted in accordance with ethical standards approved by the Animal Welfare Ethics Review Board at the University of Bradford, and in accordance with the UK National Cancer Research Institute Guidelines for the Welfare of Animals [36]. Throughout the study, all mice were housed in air-conditioned rooms in facilities approved by the United Kingdom Home Office to meet all current regulations and standards. All procedures were carried out under a Project Licence (PPL 40/3670) issued by the UK Home Office according to government legislation.

### Cell culture

H1299 and HCC193 cell lines were purchased from the ATCC. H1975 cells were obtained from the CRUK cell line bank. All cell lines were authenticated using the Human STR Profiling Cell Authentication Service at the ATCC prior to starting the present study. Cells resuscitated from liquid nitrogen storage were passaged when a confluency of 80–90% was reached, and never grown beyond 30 passages (<6 months). H1975, H1299 and HCC193 cells were grown in RPMI-1640 supplemented with 10% fetal bovine serum (FBS), 2 mM L-glutamine, 100 U/ml penicillin and 0.1 mg/ml streptomycin and maintained at 37°C in a humidified atmosphere with 5% CO2. All cell culture reagents were purchased from Sigma (Poole, Dorset, UK). All experiments were performed in the presence of 10% FBS.

### Drugs and inhibitors

CHIR99021 was purchased from A Chemtek Inc. (Worcester, MA, USA) and paclitaxel from Sigma (Poole, Dorset, UK). The drugs were dissolved in dimethyl sulfoxide (DMSO), aliquoted and stored at -20°C. For working concentrations, drugs were further diluted in DMSO and/or RPMI-1640 medium. The maximum volume of DMSO in cell culture was always <0.1%.

### Cell growth assays

Cells were seeded at 1,000 cells/per well of a black coated, clear-bottomed 96-well plate (Greiner; Stonehouse, Gloucestershire, UK) and allowed to settle overnight. Medium from cell-containing plates was gently aspirated and replaced with 100 μl of drug-containing medium. For the control, cells were exposed to medium containing DMSO only. Cells were grown in the presence of the drug(s) for 96 hours in biological triplicates, under standard cell culture conditions. At 96 hours, cells were gently washed in PBS and fixed in 4% paraformaldehyde for 30 minutes. To each well 100μl of crystal violet solution (0.05% crystal violet (Sigma) in 20% ethanol) was added and incubated at room temperature for 30 minutes. Following PBS washes, the crystal violet stain was solubilized on plate shaker for 1 hour in 100μl of 1% sodium dodecyl sulphate (Sigma; Poole, Dorset, UK) solution in PBS. Absorbance was measured at 595nm on a Perkin Elmer Fusion α plate reader and raw data analysed with Microsoft Excel.

### shRNA knockdown of GSK3

SMARTvector lentiviral shRNAs against GSK3α and GSK3β, including a pooled non-targeting shRNA control, were purchased from Dharmacon (GE Lifesciences; Buckinghamshire, UK). shRNA transductions were carried out according to the manufacturer’s instructions. Briefly, H1975 cells were seeded at 1,000 cells per well of a 96 well plate 24 hours prior to transduction. Cells were serum starved for 2 hours prior to addition of lentiviral shRNAs at an MOI of 20 in the presence of 8 μg/ml polybrene. For combined knockout of GSK3α and GSK3β isoforms, the respective shRNA sequences were pooled and added to the cells. All knockdowns were carried out in triplicate. After 96 hours, 10% v/v AlamarBlue (Thermo Fisher Scientific; Waltham, MA, USA) was added to each well. Cell viability was taken as a measurement of fluorescence at 535nm excitation and 585nm emission on a Perkin Elmer Fusion α plate reader and data analysed with Microsoft Excel. Western blot analysis was undertaken by aspirating the AlamarBlue-containing medium, washing cells with PBS, lysing cells in the well and pooling sample replicates together.

### Western blotting

Cells were lysed and western blotting undertaken as previously described [20]. Briefly, cells were lysed in 1X Lysis Buffer (Cell Signaling; Hitchin, Herts, UK) and protein concentration determined with a Pierce BCA Protein Assay Kit (Thermo Fisher Scientific; Waltham, MA, USA). Primary antibodies used were: pCRMP2 (Abcam; Cambridge, UK), ATP5ase (Santa Cruz Biotechnology; Santa Cruz, CA, USA), α-Tubulin (Sigma; Poole, Dorset, UK) and total GSK3α/GSK3β (Cell Signaling; Hitchin, Herts, UK). All primary antibodies were incubated overnight at 4°C. For standard western blot, HRP conjugated secondary anti-mouse (Jackson ImmunoResearch Laboratories; West Grove, PA, USA) or anti-rabbit (Cell Signaling; Hitchin, Herts, UK) antibodies were used. For fluorescent quantification of blots, Alexa Fluor488- and Alexa Fluor594-conjugated anti-mouse and anti-rabbit secondary antibodies were used (Thermo Fisher Scientific; Waltham, MA, USA). Blots were either developed by film or fluorescent signal measured on a Licor Odyssey machine, as appropriate.

### Immunofluorescence

H1975 cells were seeded at 1000 cells/ well of an 8 well Nunc Lab-Tek Chamber slide (Sigma; Poole, Dorset, UK), allowed to settle overnight before being treated with 2 μM CHIR99021, 1 nM paclitaxel or 2 μM CHIR99021 in combination with 1 nM paclitaxel for 72 hours. Cell medium was then aspirated, washed in PBS and fixed with ice-cold methanol for 5 minutes at -20°C. Following aspiration of the methanol, cells were brought to room temperature in PBS before permeabilisation in 0.1% Triton X100 (in PBS). A blocking step in 0.1% Triton X100 + 5% bovine serum albumin (BSA) (Sigma; Poole, Dorset, UK) preceded incubation with primary antibodies at room temperature. Primary antibodies used were rabbit anti-γ-Tubulin [1:1000] (Sigma; Poole, Dorset, UK) and DM1A mouse anti-α-Tubulin [1:250] (Sigma; Poole, Dorset, UK). Cells were then washed prior to incubation with secondary antibodies (in block): goat anti-mouse Alexa Fluor 488 and goat anti-rabbit Alexa Fluor 594 (both from Thermo Fisher Scientific; Waltham, MA, USA), both used at 1:5000. Nuclear staining was carried out with DAPI [1:10000]. Finally, the plastic chamber was carefully removed and coverslips mounted with Mowiol 4–88 mounting medium (Sigma; Poole, Dorset, UK). Images were taken at 63X magnification (NA = 1.4) on a Leica SP5 confocal microscope.

Chromosomal distribution was measured in fluorescence images using a custom MATLAB script. For each cell, fluorescently-labelled centrosomes were identified either using the Laplacian of Gaussian spot detection module of TrackMate [37], or in cases where no centrosomal marker was visible, using manual annotation based on a separate microtubule fluorescent marker. Intensity measurements for Hoescht-stained chromosomes were taken at regular intervals along the inter-centrosomal axis, with the measurement range extending to a total length twice that of the inter-centrosomal distance. Each intensity measurement was taken as the mean intensity along a line passing perpendicular to the inter-centrosomal axis, up to 7.5°μm either side of this axis. The percentage of the total chromosomal intensity detected in the non-inter-centrosomal regions was calculated for each cell.

### Xenograft studies

Male Balb/c immunodeficient nude mice (Envigo, Loughborough, U.K.), between the ages of 6 and 8 weeks were used and the H1975 non-small cell lung carcinoma model was selected for these studies. Mice were housed in cages not exceeding the numbers according to UK Home Office regulations, and were provided with bedding and nesting material and perspex housing. They were provided with food (Teklad 2018 diet, Envigo, UK) and water *ad libitum*. Animal distress and suffering were minimized by the administration of analgesics and the determination of the MTD of the compound prior to commencement of the efficacy study. Under brief general anaesthesia, 2-3mm^3^ fragments of H1975 tumor taken from donor xenografts were transplanted subcutaneously in the abdominal flanks of the efficacy study mice. Once tumor volumes reached approximately 32 mm^3^ (as measured by callipers, designated treatment day 0) mice were randomised into one of six groups (n = 8) as follows: CHIR99021 at either its maximum tolerated dose (MTD) of 75mg/kg, or half MTD of 37.5mg/kg when administered twice daily by oral gavage on days 0–3, 6–10, 13–17 and 20; paclitaxel at either its maximum tolerated dose (MTD) of 20mg/kg, or half MTD of 10/kg when administered as a single intraperitoneal dose on day 0; a combination of CHIR99021 at 37.5mg/kg per dose, plus paclitaxel at 10mg/kg (schedules as for monotherapies); untreated control. Two additional groups received maximally tolerated doses of each compound (20 mg/kg paclitaxel and 75 mg/kg CHIR99021) as monotherapy. Tumor volume, using callipers, and animal body weight were recorded throughout the experiment and normalised to the respective volume on the initial day of treatment (day 0). While each group initially consisted of 8 mice, the final numbers per group at the end-point varied between groups, with animals culled because of tumor size reaching the ethical limit. Mice were euthanized before tumors reached the maximum permissible longest diameter of 17mm. Animals were visually monitored daily by the technical staff, and tumors and bodyweight measured 5 times per week. Criteria to assess animal health were based on a combination of: ‘Pain and distress in laboratory rodents and lagomorphs’ [38] and ‘Guidelines for the welfare and use of animals in cancer research’ [36]. Mice were culled by a UK Home Office Schedule 1 (cervical dislocation) method of sacrifice. Remaining animals were culled 90 minutes following the final treatment on day 20, tumors imaged, then removed and snap frozen for further analyses. For western blot analysis, tissues were homogenized in 1X Lysis Buffer (Cell Signaling; Hitchin, Herts, UK) using a 21G needle and syringe, followed by sonication and centrifugation at 4°C. Cell supernatant was then removed and stored at -20°C. Expression of GSK3 and pCRMP2 proteins from tumor tissues was carried out as previously described.

### Statistical analysis

**Fig 1:**
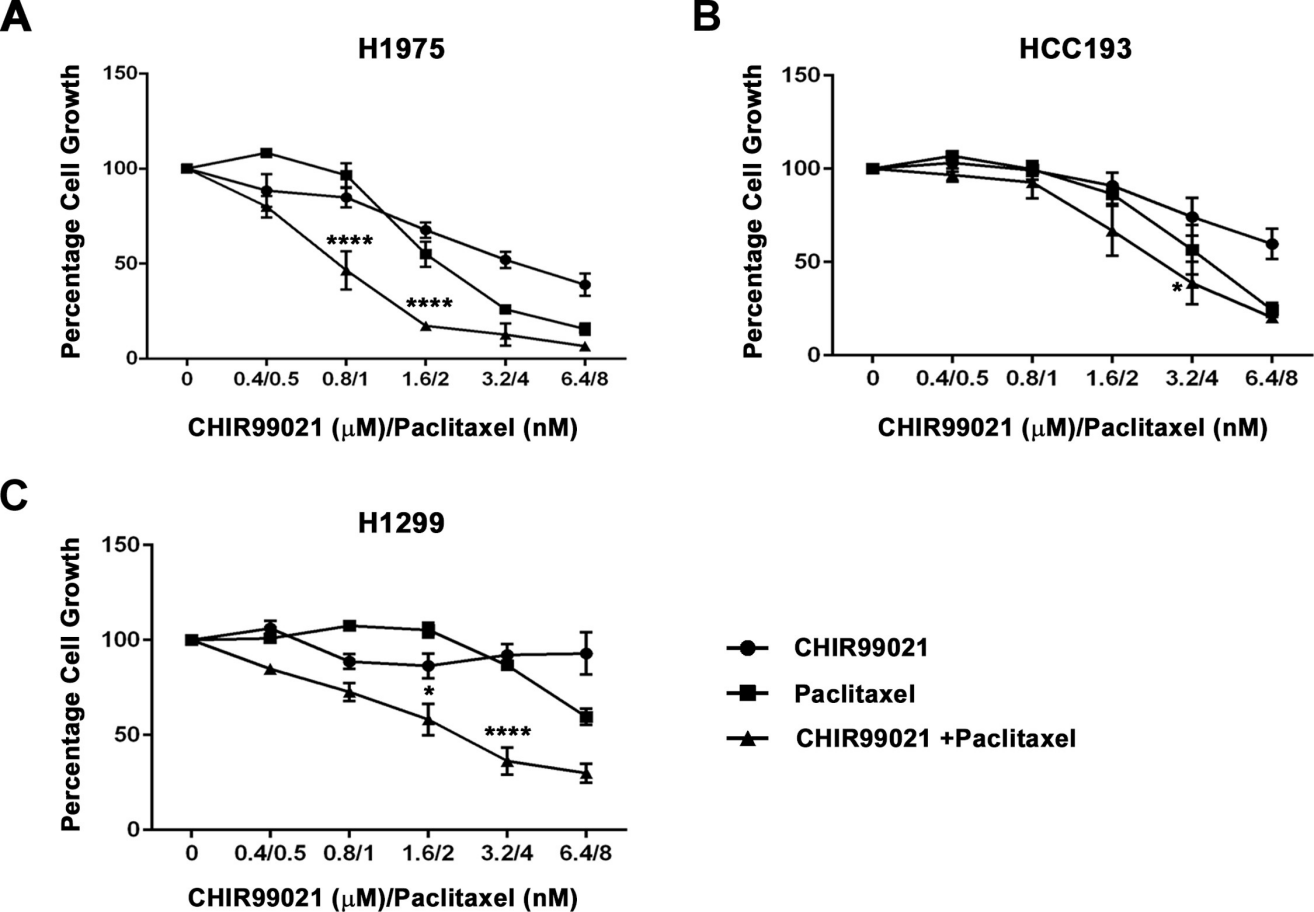
NSCLC cell lines treated with CHIR99021 and paclitaxel, as mono-treatments or in combination, show varying degrees of sensitivity. Cell growth was measured in the following NSCLC cell lines: **(A)** H1975, **(B)** HCC193 and **(C)** H1299. Cells were treated for 96 hours with 2-fold increasing concentrations of CHIR99021 (μM), paclitaxel (nM) or CHIR99021 with paclitaxel at a constant ratio of 1:800 paclitaxel to CHIR99021, respectively. A significant reduction in H1975 cell growth was observed when treated with the combined drugs at the following concentrations: 0.8 μM CHIR99021 + 1 nM paclitaxel (**** p<0.0001, 1nM paclitaxel *vs* control *vs* 0.8μM CHIR99021) and 1.6 μM CHIR99021 + 2 nM paclitaxel (**** p<0.0001, 2nM paclitaxel *vs* control *vs* 1.6μM CHIR99021). Similarly, significant responses were observed in H1299 cells treated with 1.6 μM CHIR99021 + 2 nM paclitaxel (* p<0.05, 2nM paclitaxel *vs* control *vs* 1.6μM CHIR99021) and 3.2 μM CHIR99021 + 4 nM paclitaxel (**** p<0.0001, 4nM paclitaxel *vs* control *vs* 3.2μM CHIR99021). HCC193 cells showed only a moderate response to the combined drug treatment (* p<0.05). Data are means ± standard deviation (n = 3–4). Two-way ANOVA with a Bonferroni multi comparison test was used to determine statistical relevance.

**Fig 2:**
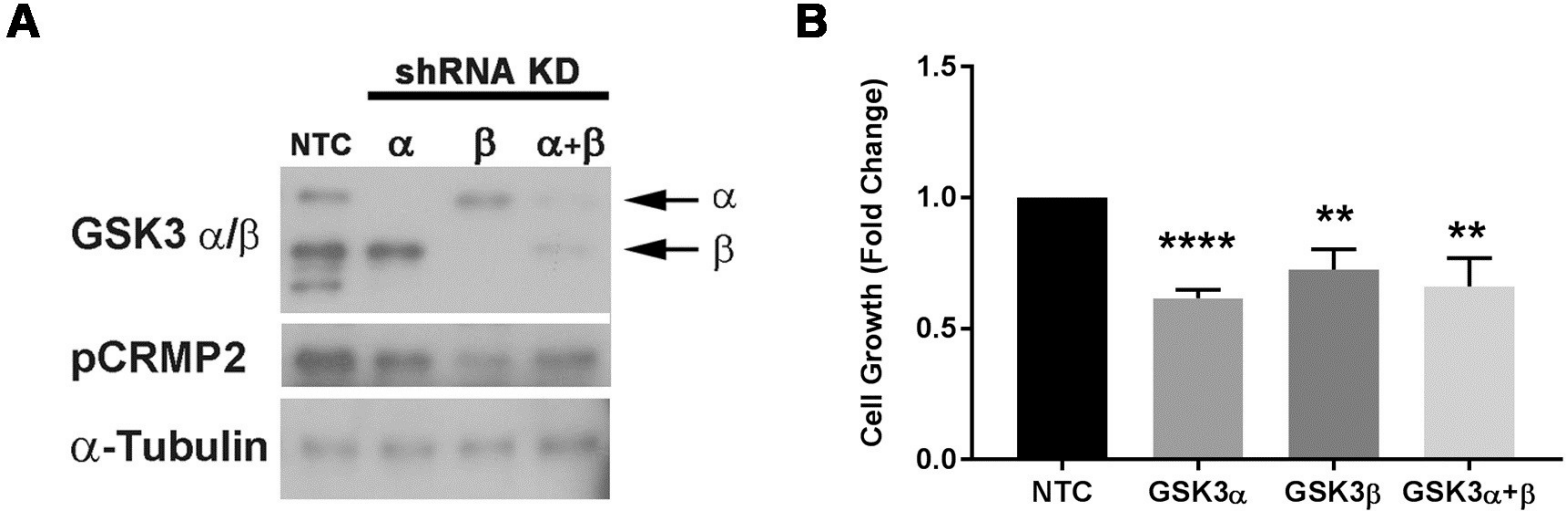
Targeted knockdown of GSK3 by shRNA reduces H1975 cell growth. In **Panel A** western blot analysis confirms an shRNA-mediated reduction in protein expression of the GSK-3α and GSK-3β isoforms in H1975 cells transduced with the GSK3α+GSKβ pooled shRNA, compared to H1975 cells transduced with a non-targeting control (NTC) shRNA (a representative blot from three separate experiments is shown). In **Panel B** H1975 cells stably transduced with shRNA against GSK3α, GSK3β and pooled anti-GSK3α and -GSK3β shRNA (GSK3α+GSKβ) showed significant reduction in cell growth. The data provide the mean ± standard deviation (n = 3) and statistical relevance was determined by standard *t*-test (** *p*<0.01 and **** *p*<0.0001 versus the non-transfected control [NTC]).

**Fig 3:**
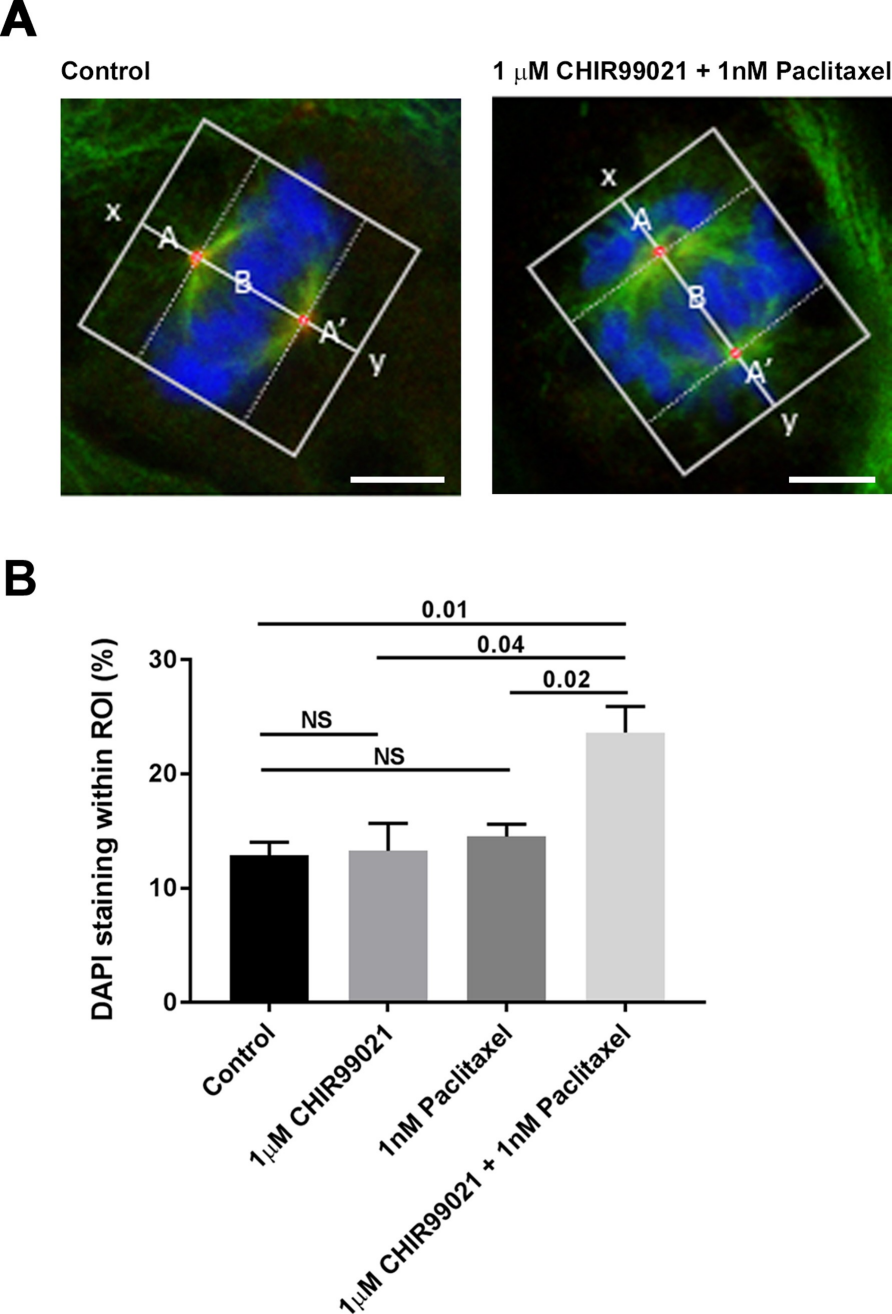
CHIR99021 and paclitaxel induce chromosomal misalignment on the metaphase plate in a synergistic manner. H1975 cells were incubated in DMSO, 2 μM CHIR99021, 1 nM paclitaxel or 2 μM CHIR99021 plus 1 nM paclitaxel for 72 hours. Cells were fixed and co-stained with DAPI, anti-γ-tubulin and anti-α-tubulin antibodies. **Panel A** shows examples of two cells which were imaged by confocal microscopy and the method by which the distribution of chromosomal DNA along an axis connecting the centrosomes of metaphase cells (x-y) was calculated using a custom MATLAB script. The amount of DAPI staining that appears within the two regions of interest (ROI) denoted A and A’ was calculated as a percentage of the total DAPI staining within ROIs A, B and A’ (i.e. 100*(A+A’)/(A+A’+B)). The scale shown bar is 5 μm. In **Panel B**, the percentage of DAPI staining lying within ROIs A+A’ provides an indication of chromosomal misalignment which is plotted as mean ± SEM for each treatment condition (three independent experiments comprising a total of 19 (control), 20 (CHIR99021), 16 (paclitaxel) and 21 (CHIR99021 + paclitaxel) data points for each condition). The figures provided above the bars provide *p* values for a two-tailed t-test for each comparison (NS = not significant).

**Fig 4:**
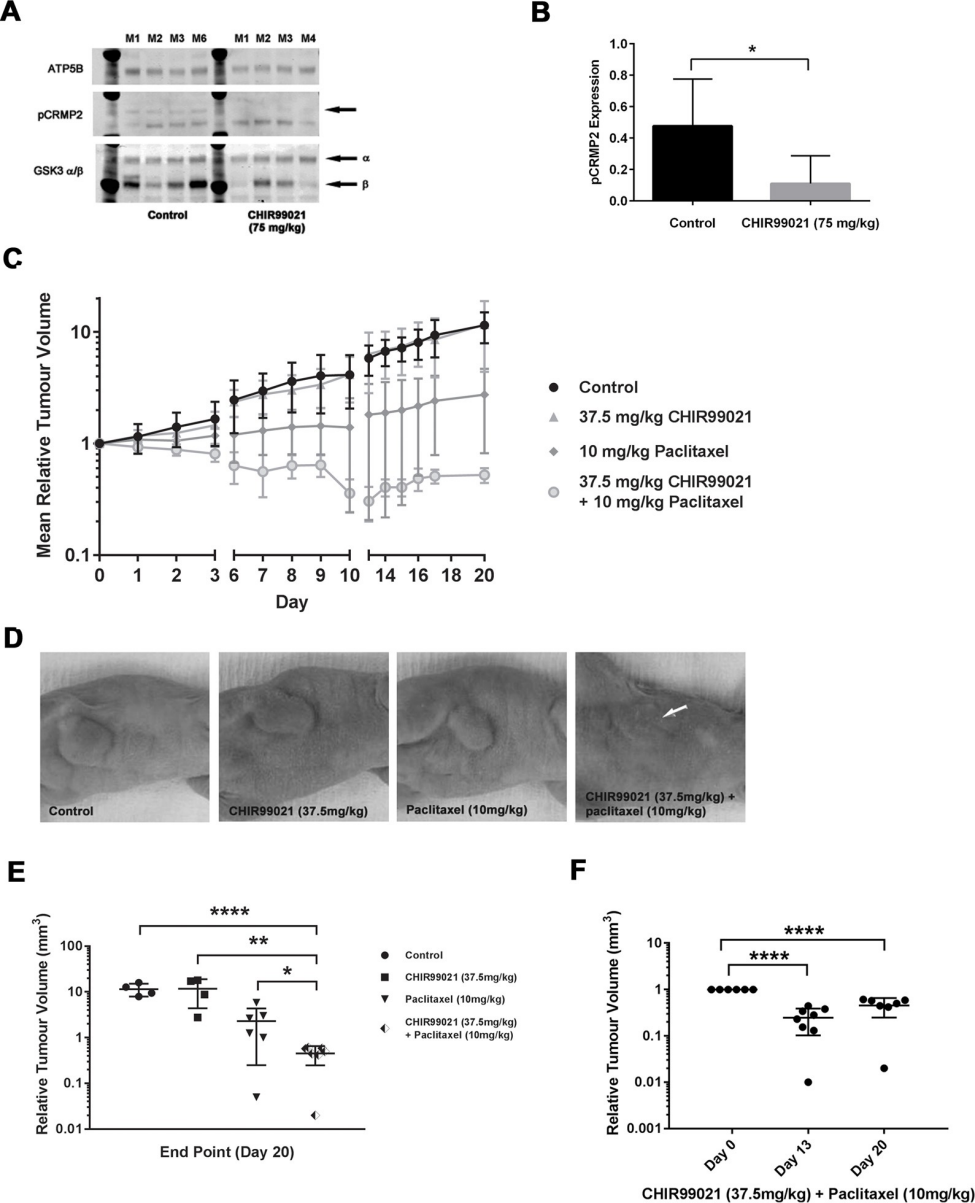
Combined treatment with CHIR99021 and paclitaxel suppresses H1975 tumor growth *in vivo*. In **Panel A**, *in vivo* inhibition of GSK3 activity by CHIR99021 was confirmed by western blot analysis of the phosphorylation status of the GSK3 substrate, CRMP2, in a panel of representative H1975 tumor tissues analysed at day 20 **(Panel A)**. The arrow in the pCRMP2 blot indicates the pCRMP2 band. **Panel B**, provides ImageJ quantification of the western blots and reveals a significant decrease (*p*<0.05) in CRMP2 phosphorylation in CHIR99021 (75 mg/kg) *vs*. control (levels were normalized to the ATP5B loading control). In **Panel C**, H1975 tumors were implanted in the right hind limb of Balb/C athymic nude mice and administered with or without a single intraperitoneal half-maximally tolerated dose of paclitaxel, with or without an additional half-maximally tolerated oral dose of CHIR99021 twice daily every five days for three weeks, with a two day treatment holiday between each five day regimen (n = 8 mice per treatment group). Reduction in tumor volume was observed for the mice treated with 10 mg/kg paclitaxel; however, no effect was noted in the 37.5 mg/kg CHIR99021 cohort. Strikingly, the combination of 37.5 mg/kg CHIR99021 with 10 mg/kg paclitaxel substantially attenuated tumor growth. In **Panel D**, representative images of end-point tumors taken from mice in each cohort. The white arrow indicates the location of each tumor, which fully recedes following treatment with the two drugs in combination. **Panel E**, provides the median tumor size which was analysed at the study end-point (n = 4–7 per group) with *p* values of *p*<0.0001 (****), 0.002 (**) and 0.04 (*) indicated. In **Panel F**, we compared the effect of treatments on tumor volume at day 13 and day 20 versus that at day 0 (mean ± standard deviation is shown; **** indicates *p*<0.0001). No significant difference was found between day 13 and day 20 tumor volume.

Statistical analyses were performed using GraphPad Prism. For *in vitro* viability assays, two-way ANOVA with a Bonferroni multiple comparison tests were performed (Fig 1). A two-tailed student *t*-test was performed for analysis of chromosomal segregation (Figs 2 and 3) and for the analysis of the xenograft study data (Fig 4 panels E and F). Unless otherwise stated, all experiments were derived from a minimum of three independent experiments. Error is represented as mean ± standard deviation (s.d.).

## Results

### In vitro inhibition of growth

In this study we examined three NSCLC cell lines (H1975, H1299 and HCC193) for the effects of CHIR99021 on cell growth when used alone or in combination with the chemotherapeutic agent, paclitaxel. As shown in Fig 1, we observed a subtle variety of responses of these cell lines to both compounds.

H1975 and HCC193 cells were sensitive to paclitaxel across the 2–8 nM dose range with 8 nM drug causing almost complete cessation of growth (Fig 1 panels A and B, respectively). By contrast, H1299 cells only showed sensitivity at the two highest paclitaxel doses used (4 and 8 nM; Fig 1 panel C). Consistent with our previously reported observations for CHIR99021 [20], the growth of H1975 and HCC193 cell lines were both significantly reduced by CHIR99021 (p<0.0001 and p<0.05 compared to control, respectively; Fig 1 panels A and 1B). However, the growth of H1299 cells was relatively insensitive to CHIR99021 (Fig 1 panel C).

We next explored whether the two drugs used in combination at the same series of escalating doses, acted additively or synergistically on cell growth. The dose response curves for H1975 and H1299 cells both demonstrated evidence for a synergistic inhibition of cell growth in the presence of both drugs in combination, relative to that expected from simply adding together the effect of each compound when used individually (Fig 1). For example, H1975 cells demonstrated very little inhibition of growth at either 0.8 μM CHIR99021 or 1 nM paclitaxel as individual treatments (15.3 ± 6.8% and 3.6 ± 8.0% inhibition for each dose, respectively; mean ± standard error of mean (SEM) for three independent experiments), however the combination of the two drugs reduced growth by 53.5% ± 11.8% inhibition of growth (p = 0.015 for the difference between the observed effect of the combination versus the predicted effect through adding the individual effects of each compound). Similarly, for the H1299 cell line 3.2 μM CHIR99021 plus 4 nM paclitaxel had relatively little effect when used individually (7.8 ± 13.7% and 13.3 ± 7.3% inhibition for each dose, respectively; mean ± SEM for four independent experiments), but in combination brought about a 63.7 ± 16.5% inhibition of growth (p = 0.005 for the difference between observed versus predicted effects).

### shRNA-mediated knockdown of GSK3α and GSK3β inhibits proliferation of H1975 cells

To confirm that GSK3 was contributing to the growth of NSCLC cells, we deployed a shRNA approach to stably knockdown GSK3α and GSK3β isoforms, both individually and together in H1975 cells. We confirmed the isoform-selective knockdown of GSK3α and GSK3β expression levels and consequent reduction in GSK3 activity as determined by measuring the phosphorylation of CRMP2, a highly selective GSK3 substrate [4](Fig 2 panel A). Moreover, the reduction in each isoform caused a significant inhibition of growth (approximately 40%) compared to the effect of the non-targeting control shRNA (Fig 2 panel B). In attempting to knock-down the expression of both isoforms, we combined the two shRNAs but were unable to achieve complete knock-down of both GSK3α and GSK3β (Fig 2 panel A).

The knockdown of GSK3α together with GSK3β decreased cell growth to a similar extent as knock-down of GSK3β alone, which is consistent with the fact that the total combined amount of GSK3α and GSK3β protein and protein kinase activity (measured as CRMP2 phosphorylation) remaining in the cells was similar to that present in GSK3β knockdown cells (Fig 2 panel A). The data suggest that GSK3α and GSK3β are both important in controlling cell proliferation in H1975 cells and that only relatively small amounts of the two enzymes are required to maintain cell growth. The data further suggest that the mechanism by which CHIR99021 inhibits growth is via its ability to inhibit GSK3α/β although, despite it being the most selective ATP-competitive GSK3 inhibitor available [32], we cannot exclude the possibility that at least some aspects of its action are the result of effects on kinases or other ATP-dependent proteins not related to GSK3.

### CHIR99021 and paclitaxel promote chromosome misalignment on the metaphase plate in a synergistic manner

To explore the mechanism by which GSK3 contributes to cell proliferation, we examined the effects of CHIR99021 and paclitaxel on chromosomal alignment in metaphase cells. H1975 cells were treated with 1 μM CHIR99021, 2 nM paclitaxel (sub-maximally effective doses for growth inhibition; Fig 1 panel A) or a combination of 1 μM CHIR99021 plus 2 nM paclitaxel. After 72 hours, the cells were then fixed and stained with antibodies against γ-tubulin and α-tubulin antibodies which are markers for spindle centrosomes and microtubule structures, respectively and with DAPI to label chromosomal DNA. All cells that had entered a state of at least partial chromosome alignment on the metaphase plate were identified, imaged by confocal microscopy and chromosomal alignment quantitatively analysed (Fig 3 panel A) (representative images of all treatment groups are shown in S1 Fig).

As shown in Fig 3 panel B, CHIR99021 and paclitaxel, added alone at 1 μM or 2 nM respectively, had no significant effect on the alignment of chromosomes on the metaphase plate. By contrast, the combination treatment promoted a highly significant increase in the percentage of misaligned chromosomal DNA in a synergistic manner relative to the DMSO control or cells treated with CHIR99021 or paclitaxel on their own (*p* = 0.01, 0.04 and 0.02 versus each of DMSO, CHIR99021 or paclitaxel alone).

### In vivo inhibition of growth

To investigate whether combined treatment with CHIR99021 and paclitaxel had a synergistic effect on tumor growth in an *in vivo* situation, H1975 tumors were established in Balb/C athymic mice and treated with CHIR99021 and/or paclitaxel as monotherapies or in combination (Figs 4 and S2). Neither compound, used alone or in combination, had an adverse effect on animal weight during the treatment period which lasted for up to 20 days (S2 Fig).

CHIR99021 is orally bioavailable but has a relatively short half-life in plasma, hence it was administered twice daily by oral gavage. To confirm that CHIR99021 was both capable of penetrating tumor tissue and effectively at inhibiting tumor GSK3 activity, we measured the level of phosphorylated CRMP2 as a proxy of *in vivo* GSK3 activity. Representative blots of tumors derived from four animals within each of the control and CHIR99021 treated groups is shown in Fig 4 panel A. Compared to the control animals, a significant reduction of CRMP2 phosphorylation was apparent in animals treated with 75mg/kg CHIR99021 (*p*<0.05; Fig 4B) confirming the target efficacy of the compound under the treatment regimen used.

In contrast to our *in vitro* findings, we observed no reduction in tumor volume when CHIR99021 was administered as a monotherapy (Fig 4 panel C). On the other hand, 10 mg/kg paclitaxel monotherapy significantly reduced tumor volume (*p*<0.001, Day 20; Fig 4 panel C). A further, more pronounced, reduction in tumor growth was seen at the higher dose of paclitaxel (20mg/kg; S3 Fig).

Mice treated with the combination of 37.5mg/kg CHIR99021 and 10mg/kg paclitaxel (half-maximally tolerated doses of each individual compound) showed a significant reduction in tumor volume was observed compared to that with 10mg/kg paclitaxel administered alone (Fig 4C and 4D). These observations confirm that paclitaxel and CHIR99021 act in a synergistic fashion to inhibit H1975 tumor growth *in vivo*, consistent with the results of the *in vitro* experiments with H1975 cells (Fig 1 panel A). Furthermore, we also showed a reduction in tumor volume in the combined treatment over the course of the experiment (*p*<0.0001, day 13 and day 20 compared to day 0) (Fig 4 panel F).

## Discussion

Despite numerous advancements in treatments for NSCLC, especially in the field of targeted therapies, the rate of overall survival in patients with late stage disease remains unacceptably low. To combat the challenge of emerging resistance, novel targetable biomarkers and therapeutic treatments need to be explored. In this study we evaluated the use of a small-molecule GSK3 inhibitor, CHIR99021, in combination with paclitaxel in inhibiting the growth of NSCLC cells. Paclitaxel is a 1st generation taxane which showed a 40% increase in 1-year survival rate in advanced and metastatic NSCLC patients [39]. These promising results, as a single-agent, have prompted numerous studies into combination of paclitaxel with other agents, in particular platinum-based compounds (e.g. carboplatin and cisplatin), and its inclusion in standard regimens for the treatment of NSCLC. Here we provide evidence that paclitaxel and CHIR99021 act in a synergistic manner to both prevent chromosomal alignment during mitosis and to inhibit the growth of NSCLC cells *in vitro* and in a mouse tumor xenograft model.

In the tumor xenograft model, we examined the efficacy in inhibiting tumor growth of orally-administered daily dosing of CHIR99021 together with a single bolus of intraperitoneally-delivered paclitaxel (Fig 4). In this *in vivo* model, CHIR99021 was without effect on tumor growth when used on its own. By contrast, paclitaxel promoted an inhibition of tumor growth at a half-maximally tolerated dose, and this was substantially and significantly enhanced in the presence of CHIR99021 (Fig 4). Indeed, we also found a reduction in tumor volume in the combined treatment over the course of the experiment which could be a consequence of reduced cell proliferation with a maintained background of cell death or increased cell death with a maintained background of cell proliferation. To discriminate between these mechanisms, or whether it involves a combination of both, will require future investigation using immunostaining tumors with apoptotic (cytokeratin 18 cleavage) or proliferation (Ki67) markers. Such a reduction in tumor burden could be of significant patient benefit.

Our xenograft results corroborate the striking synergistic effect we observed *in vitro* (Fig 1), although contrary to the xenograft model, H1975 cells grown in culture demonstrate a significant inhibition of cell growth in response to CHIR99021 when used alone (Fig 1). One explanation for this difference may be attributed to the more complex regulation of tumor growth in an *in vivo* environment.

We previously demonstrated that CHIR99021 also stabilises mitotic microtubules and promotes misalignment of chromosomes on the metaphase plate [33]. Interestingly, this effect was reported to be mediated through the ability of GSK3 to phosphorylate CRMP4 [40], which is related to CRMP2 used in our own study to measure GSK3 activity (Figs 2 and 4). We thus investigated the relationship between growth inhibition by CHIR99021 and paclitaxel, and the ability of these agents to promote defects in chromosome alignment during mitosis.

As shown in Fig 3, neither CHIR99021 nor paclitaxel when added alone had an observable effect on the alignment of chromosomes on the metaphase plate at the doses employed. This is most likely due to two things: (i) the very low doses of drugs used (sub-maximal doses, calculated from Fig 1) and (ii) the stringency of our quantitative image analysis, in which only chromosomes that are misaligned to the extremes of the spindle poles are measured. When added in combination, however, CHIR99021 and paclitaxel profoundly inhibit chromosomal alignment (Fig 3). This synergism was consistent with our observations in the *in vitro* growth experiments and may derive from the fact that the agents deploy different mechanisms to stablise spindle microtubules; paclitaxel by directly binding tubulin polymers and preventing depolymerisation [34]; CHIR99021 by inhibiting GSK3 and preventing CRMP2/4 phosphorylation [33, 40]. Taken together our results suggest that the mechanism by which these agents bring about their effect on the inhibition of tumor growth may be via microtubule stabilisation and subsequent misalignment of chromosomes during mitosis.

GSK3 phosphorylated on serine 21/9 is found on the spindle apparatus together with (i) Akt, the upstream kinase responsible for this phosphorylation [33], and (ii) phosphoCRMP4, a downstream target GSK3 substrate [40]. It will be of interest to determine whether the spindle-associated pool of GSK3 is regulated by growth factors and/or serum, or whether it defines a distinct pool of GSK3 that is CHIR99021-inhibitable but unresponsive to serum and growth factors.

Interestingly, our *in vitro* assays showed that growth reduction in certain cell lines (namely, H1975 and HCC193 cells) occurred in a dose-responsive manner to CHIR99021, whereas H1299 cells were relatively resistant to the compound (Fig 1). We previously demonstrated that the growth of PC9 cells was also inhibited by CHIR99021 [20], whereas in the current study we found that the growth of two other NSCLC cell lines (HCC95 and HCC827 cells) were also resistant to CHIR99021 (S4 Fig). The sensitivity of NSCLC cells to growth inhibition by paclitaxel was also variable; H1975 and HCC193 (Fig 1) and HCC95 (S4 Fig panel A) cells were sensitive to the compound, whereas the growth of other cells lines we examined were relatively (H1299; Fig 1) or completely (HCC827; S4 Fig panel B) insensitive. This differential sensitivity to paclitaxel is consistent with previous observations in NSCLC cell lines [41–43]. As inhibition of GSK3 with the CHIR99021 attenuates cell proliferation, we compared apoptotic induction, looking at expression of the apoptotic protein, Annexin V, in CHIR99021-sensitive (H1975) and–insensitive (H1299) cell lines. H1975 cell showed increased expression of Annexin V when treated with 5 μM and 10 μM CHIR99021 (p<0.05), whereas no change in Annexin V expression was observed in H1299 at any of the CHIR99021 concentrations tested (S5 Fig).

Our findings are in keeping with the 2013 study by Yin et al., who also demonstrated that combination of GSK3 inhibition, using the GSK3 inhibitor lithium chloride (LiCl), in combination with paclitaxel treatment in endometrial cancer cells [44]. Namely, that GSK3 inhibition in combination with paclitaxel treatment can effectively attenuate tumor growth. Our study builds on the findings of the Yin et al. paper by looking at the effects of the highly specific CHIR99021 in combination with paclitaxel, both *in vitro* and *in vivo*, and demonstrating that this particular combination has a more synergistic effect on tumor cell growth reduction in NSCLC cells sensitive to the treatment compared to the combination of LiCl and paclitaxel in the endometrial cell line ANC3A. Taken together, the two studies highlight the potential of GSK3 inhibition in the development of novel treatment regimens for a broader range of cancer indications.

That the growth of only a subset of NSCLC cell lines demonstrate inhibition by CHIR99021 is, perhaps, unsurprising since we have previously observed an over-expression and hyperactivation of GSK3 in only approximately 40% of samples of human NSLSC when compared to patient-matched surrounding normal lung tissue [20]. This raises the intriguing possibility that GSK3 hyperactivation might represent a useful biomarker for sensitivity to CHIR99021-mediated growth inhibition in patients. It seems unlikely that the increased expression of GSK3 in tumors is due to copy number variation. The 2012 Cancer Genome Atlas study showed that GSK3B was amplified at a frequency of 6.7% (12/178 profiled samples) in NSCLC patients with a squamous cell carcinoma histological subtype [45]. However, subsequent studies have suggested a lower occurrence rate for GSK3 amplification, ranging between 0.1% to 1.3% of the patient population studied [46, 47]. While the frequency at which GSK3 amplification occurs in the NSCLC population is low, it appears that histological subtype may have an influence and that this may be of importance when screening and stratifying NSCLC patients based on GSK3 levels. The increased GSK3 expression and activity may, therefore, be more related to increased GSK3 gene promoter activity or stabilisation of GSK3 protein. We are unable to distinguish between these possibilities, but they certainly warrant future investigation.

Variable sensitivity to CHIR99021 is of additional potential interest given the link between elevated GSK3 expression and poor patient prognosis, which has been reported by others in both lung and other cancer types [12–17, 23]. However, because our current study in NSCLC cell lines was insufficiently powered to confirm whether the degree of sensitivity of NSCLC cell lines to CHIR99021 is related to the level of GSK3 expression and/or activity, this possibility will require further investigation in a larger set of NSCLC cell lines. Equally, whether the sensitivity is related to the genetic background of the cells (the H1975 cell line is an EGFR T790M mutant positive cell line resistance to erlotinib, and the H1299 cell line is an N-Ras mutant cell line) will require an appropriately powered study in a larger panel of cell lines.

The level of CRMP2 phosphorylation, could provide a useful surrogate biomarker for GSK3 activity [4, 48] within a clinical trial. However, given that there may not be a not be a simple relationship between GSK3 protein, activity and phosphorylation status of a single substrate, one could measure the phosphorylation of a range of substrates such as glycogen synthase-S641 and NFkB p65-S468 phosphorylation [20]. In this way a companion diagnostic could be developed to identify those patients most susceptible to the combination treatment.

A recent phase-I trial demonstrated that a GSK3 inhibitor, LY2090314, is well tolerated in humans and it is well documented that long-term treatment with lithium (a known GSK3 inhibitor) in psychiatric patients is well tolerated [49]. It is of further interest that these same psychiatric patients have a lower incidence of cancer than the general population [49]. In our *in vivo* study, we observed no toxicities in the animals in the presence of both drugs, either in combination or when used as monotherapy (S2 Fig). Thus administration of a GSK3 inhibitor in a cancer treatment regime may be well tolerated particularly if given in combination with paclitaxel at submaximal, and synergistically acting, doses. We believe, therefore, that this drug combination has promising potential for further development as a novel therapeutic approach for treating NSCLC.

## Supporting information

S1 Fig Increased instances of chromosomal misalignments following combined treatment with CHIR99021 and paclitaxel. Representative images illustrating spindle abnormalities and chromosomal misalignments in H1975 cells grown in the presence of 1 μM CHIR99021 and 2 nM paclitaxel for 72 hours.
(TIF)

S2 Fig Animal weights during dosing regimen. Animal weights between the different treatment groups remained comparable to the control throughout the 20 day xenograft study, confirming that neither compound, administered either alone (at MTD and half MTD) or in combination at half MTD, had any adverse cytotoxic effects.
(TIF)

S3 Fig Paclitaxel suppresses H1975 tumor growth *in vivo*. Paclitaxel monotherapy reduced tumour volume at both 10 mg/kg (half-maximally tolerated dose) and 20 mg/kg (maximally tolerated dose) in a dose-dependent manner. A more pronounced effect on tumor growth suppression can be observed following treatment with 20 mg/kg paclitaxel compared to 10 mg/kg paclitaxel.
(TIF)

S4 Fig NSCLC cell lines, HCC95 and HCC827, demonstrate different sensitivities to CHIR99021 and paclitaxel treatment. In **Panel A**, the NSCLC cell line, HCC95, was shown to be resistant to growth suppression in the presence of CHIR99021, whereas growth inhibition was achieved when cells were treated with paclitaxel. HCC827 cells, **Panel B**, were found to be insensitive to treatment with either CHIR99021 or paclitaxel.
(TIF)

S5 Fig GSK3 inhibition in CHIR99021-sensitive H1975 cells induces an apoptotic response. Apoptotic induction, measured on the basis of Annexin V expression with a FITC Annexin V apoptosis detection kit (BioLegend, London, UK), was measured in the following NSCLC cell lines: H1975 and H1299, according to manufacturer’s instructions. Cells were treated for 72 hours with 2-fold increasing concentrations of CHIR99021 (μM), paclitaxel (nM) or CHIR99021 with paclitaxel, as described in Fig 1. Cells were treated in triplicate in a 96-well plate, data are presented as means ± standard deviation (n = 3 independent experiments for H1975, n = 2 for H1299). Data were normalized to cell number, determined using crystal violet staining. Increased expression of Annexin V was observed in H1975 cell at 5 μM and 10 μM CHIR99021 (p<0.05) but not no response was seen in CHIR99021-insensitive H1299 cells. Two-way ANOVA with a Bonferroni multi comparison test was used to determine statistical relevance.
(TIF)
