# Supplementary figures and images for: Tumor growth suppression using a combination of taxol-based therapy and GSK3 inhibition in non-small cell lung cancer
Source: PLoS One. 2019 Apr 10;14(4):e0214610. doi: 10.1371/journal.pone.0214610 (PMC6457575; doi:10.1371/journal.pone.0214610)

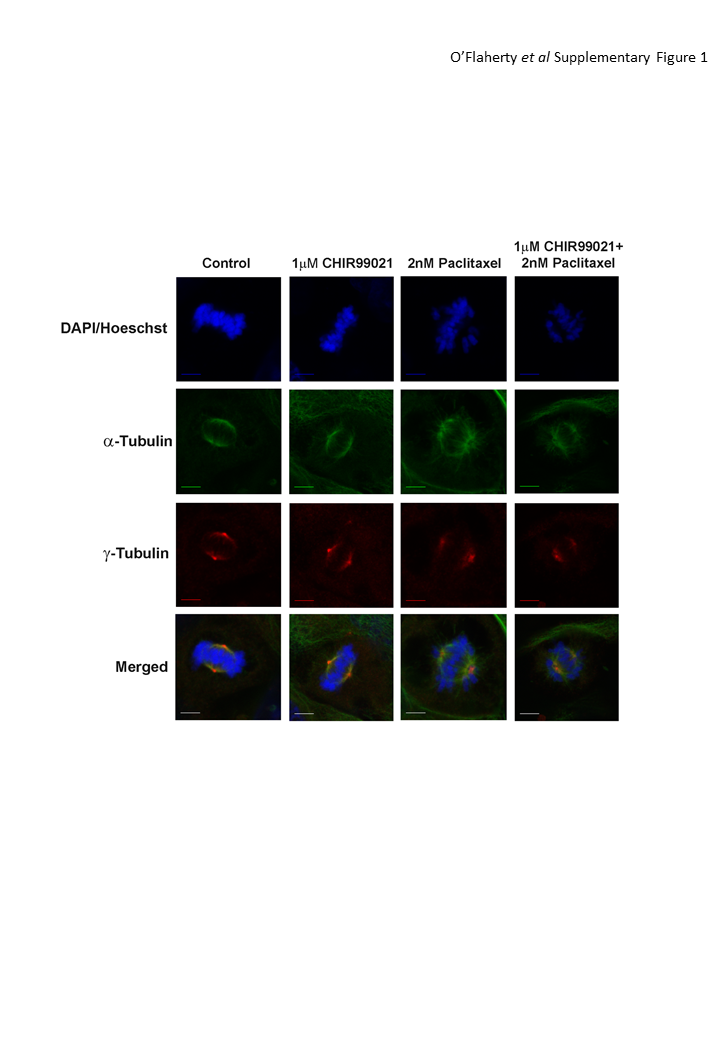

Supplement: S1 Fig — Representative images illustrating spindle abnormalities and chromosomal misalignments in H1975 cells grown in the presence of 1 μM CHIR99021 and 2 nM paclitaxel for 72 hours. (TIF) [file pone.0214610.s001.tif]

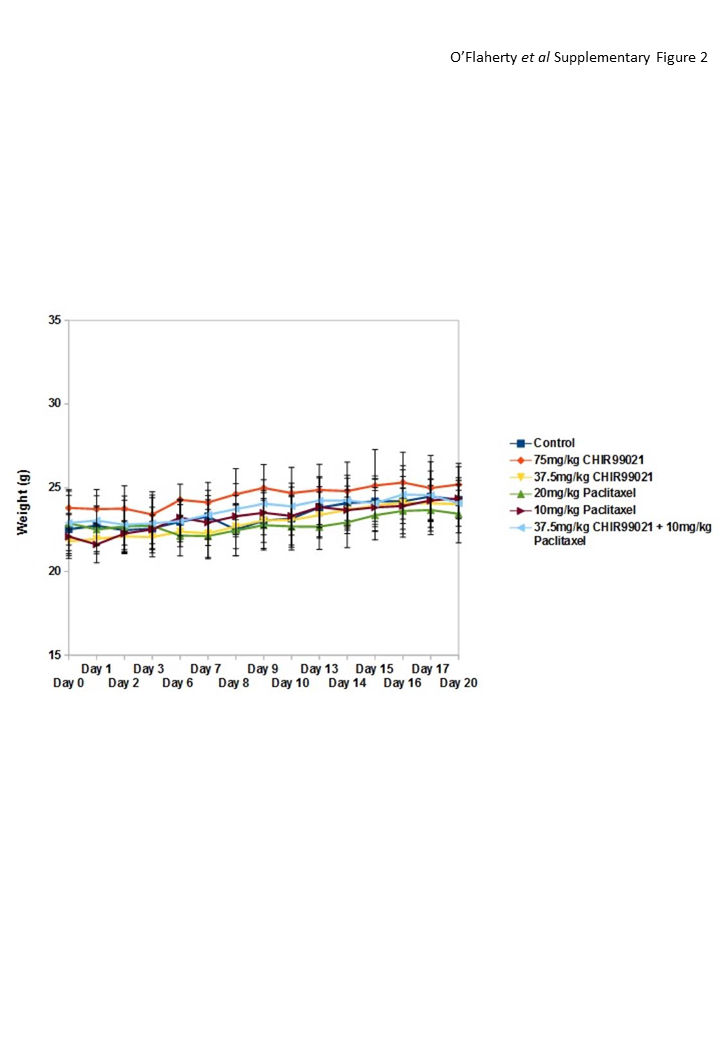

Supplement: S2 Fig — Animal weights between the different treatment groups remained comparable to the control throughout the 20 day xenograft study, confirming that neither compound, administered either alone (at MTD and half MTD) or in combination at half MTD, had any adverse cytotoxic effects. (TIF) [file pone.0214610.s002.tif]

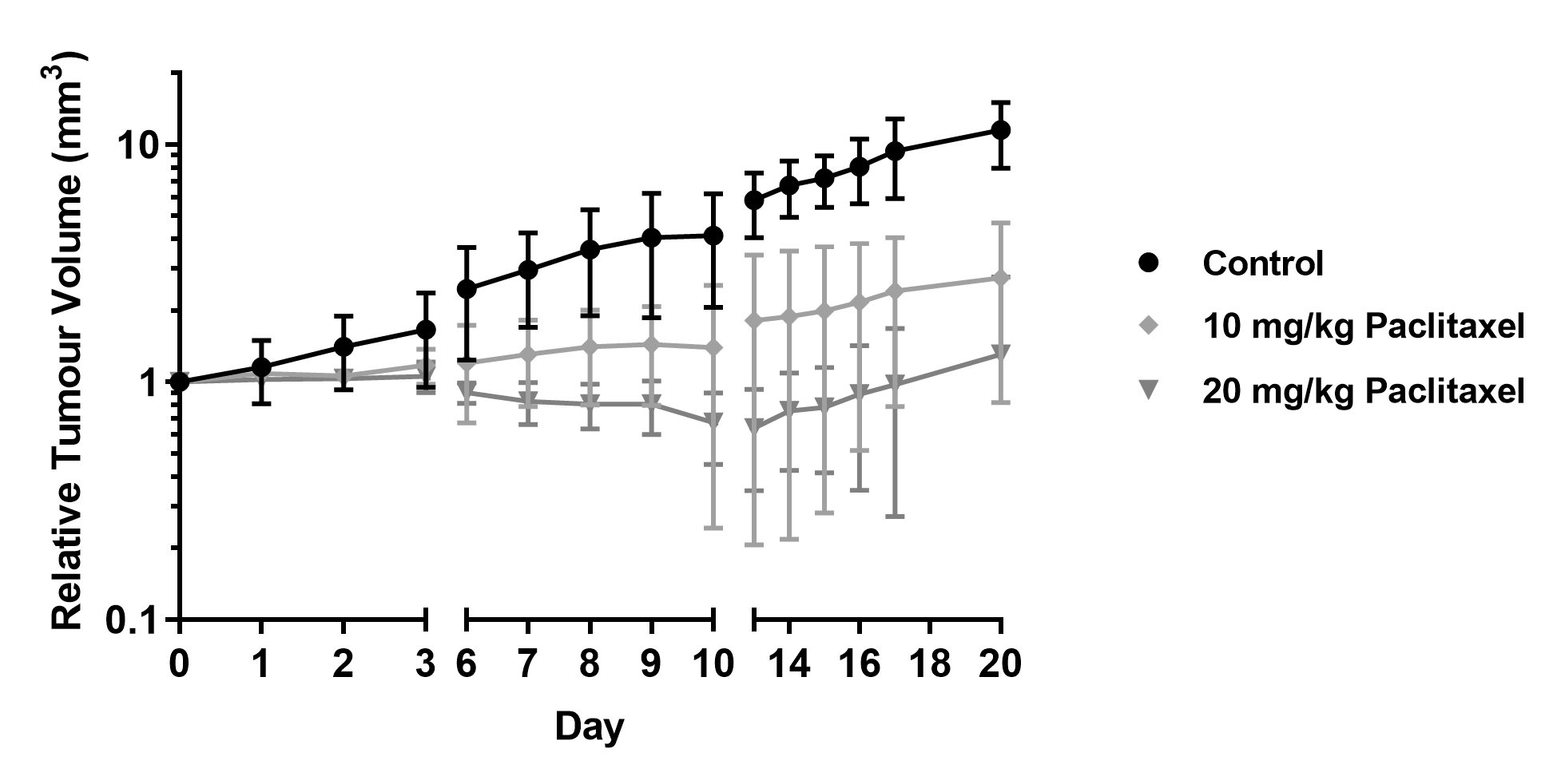

Supplement: S3 Fig — Paclitaxel monotherapy reduced tumour volume at both 10 mg/kg (half-maximally tolerated dose) and 20 mg/kg (maximally tolerated dose) in a dose-dependent manner. A more pronounced effect on tumor growth suppression can be observed following treatment with 20 mg/kg paclitaxel compared to 10 mg/kg paclitaxel. (TIF) [file pone.0214610.s003.tif]

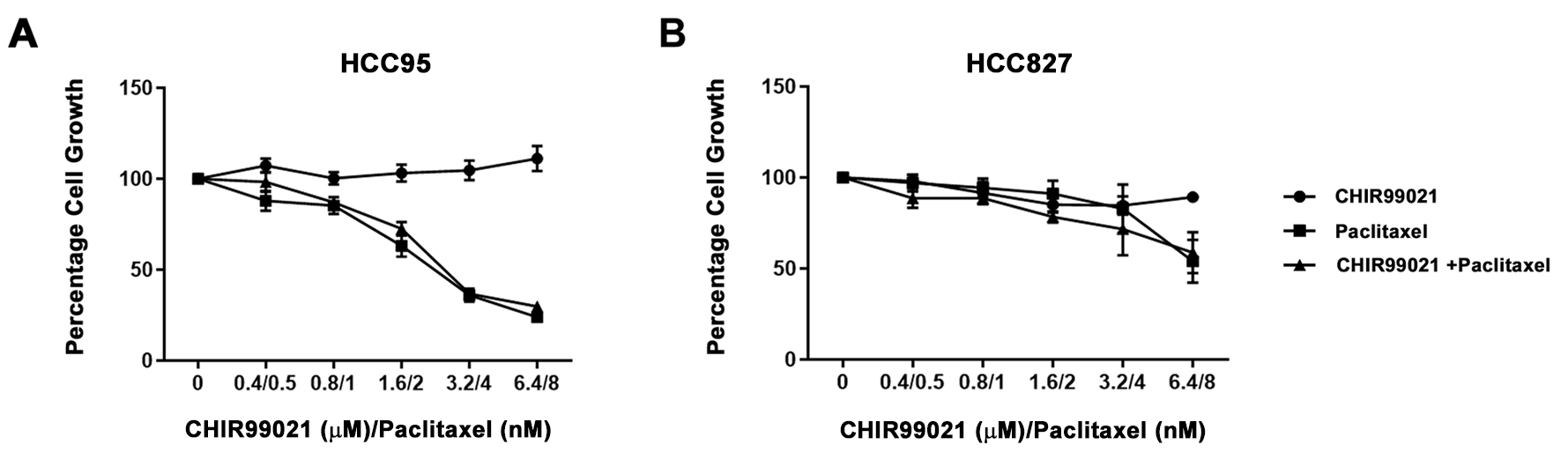

Supplement: S4 Fig — In Panel A, the NSCLC cell line, HCC95, was shown to be resistant to growth suppression in the presence of CHIR99021, whereas growth inhibition was achieved when cells were treated with paclitaxel. HCC827 cells, Panel B, were found to be insensitive to treatment with either CHIR99021 or paclitaxel. (TIF) [file pone.0214610.s004.tif]

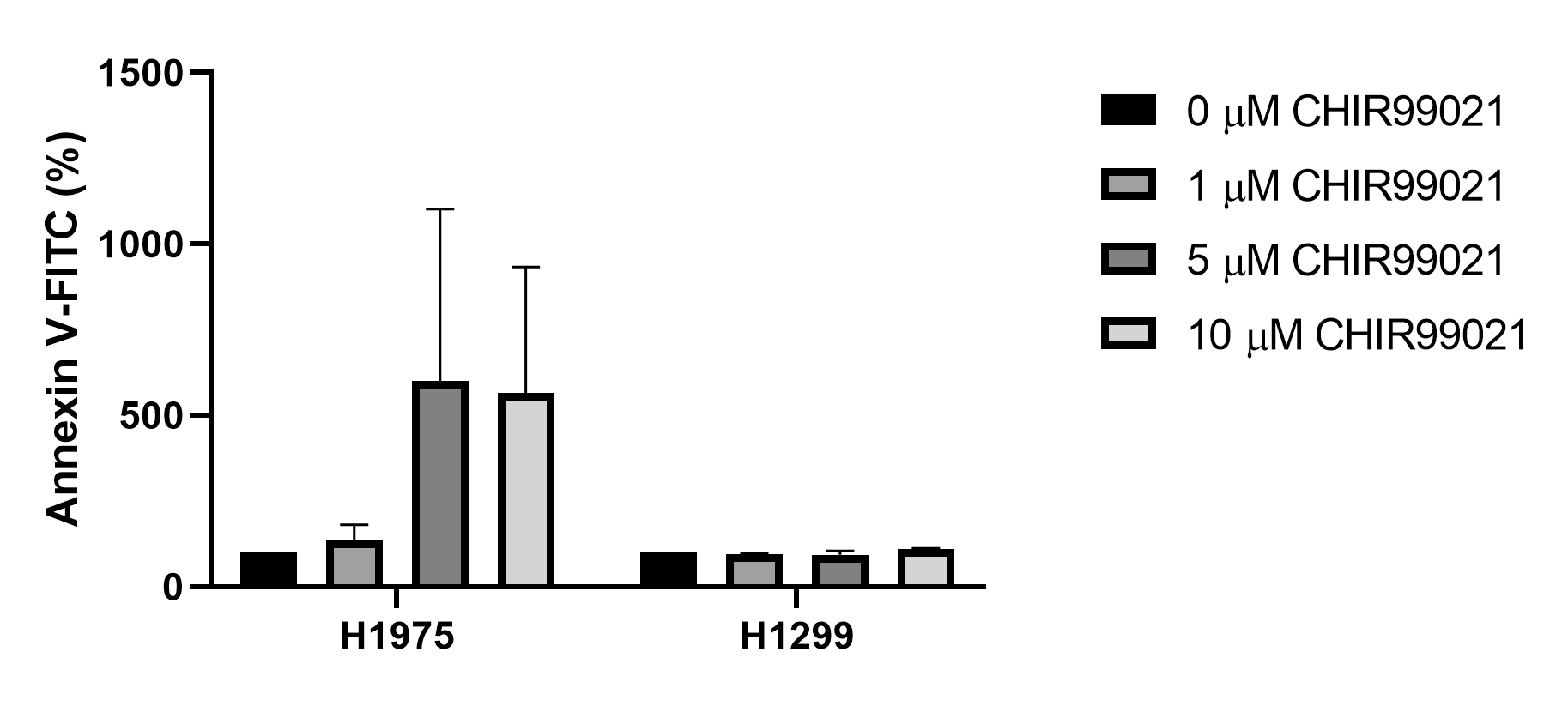

Supplement: S5 Fig — Apoptotic induction, measured on the basis of Annexin V expression with a FITC Annexin V apoptosis detection kit (BioLegend, London, UK), was measured in the following NSCLC cell lines: H1975 and H1299, according to manufacturer’s instructions. Cells were treated for 72 hours with 2-fold increasing concentrations of CHIR99021 (μM), paclitaxel (nM) or CHIR99021 with paclitaxel, as described in Fig 1. Cells were treated in triplicate in a 96-well plate, data are presented as means ± standard deviation (n = 3 independent experiments for H1975, n = 2 for H1299). Data were normalized to cell number, determined using crystal violet staining. Increased expression of Annexin V was observed in H1975 cell at 5 μM and 10 μM CHIR99021 (p<0.05) but not no response was seen in CHIR99021-insensitive H1299 cells. Two-way ANOVA with a Bonferroni multi comparison test was used to determine statistical relevance. (TIF) [file pone.0214610.s005.tif]
